# Clinical features and pathogen distributions of microbiological-based protracted bacterial bronchitis in children of different ages in Northeast China

**DOI:** 10.3389/fped.2023.1163014

**Published:** 2023-04-21

**Authors:** Ning Chen, Han Zhang, Yong Feng

**Affiliations:** Department of Pediatrics, Shengjing Hospital of China Medical University, Shenyang, China

**Keywords:** *Klebsiella pneumoniae*, protracted bacterial bronchitis, child, chronic wet cough, wheezing, bacteria

## Abstract

**Background:**

Protracted bacterial bronchitis (PBB) is often diagnosed clinically according to chronic wet cough, which can be resolved by appropriate antibiotics. Though rarely performed in PBB diagnosis, bacterial cultures by sputum or bronchoalveolar lavage (BAL) fluid can provide etiological features, which may be different in western countries and different areas of China. This study aimed to investigate the clinical and etiological features and outcomes in children of different ages with PBB in northeast China.

**Methods:**

We retrospectively analyzed children diagnosed with PBB by positive BAL fluid or sputum bacterial cultures between 2017 and 2021. Children were divided into three age groups: <1 year (infants), 1–5 years (younger children), and ≥6 years (older children). Clinical characteristics, chest radiographic findings, bronchoscopy findings, microbiological findings, treatment strategies, and outcomes were reviewed and compared among the age groups. Factors associated with remission during follow-up were examined using logistic regression.

**Results:**

A total of 45 children with PBB were included, consisting of 24 (53.3%) infants. The infants were often boys and had a shorter cough duration, a lower proportion of expectoration, a greater proportion of wheezing, and less bronchial wall thickening on high-resolution computed tomography compared to older children (*P* < 0.05). No significant differences were found among the age groups regarding macroscopic findings, except for a higher proportion of tracheobronchial malacia in infants than in older children (*P* = 0.013). The most commonly cultured bacteria were *Haemophilus influenzae* (42.2%), followed by *Streptococcus pneumoniae* (22.2%) and *Klebsiella pneumoniae* (20.0%). Compared to older children, infants had a higher remission (*P* = 0.009) and relatively lower relapse rates (*P* = 0.059). Short duration of cough (*OR *= 0.58, 95% *CI*: 0.34–0.99, *P* = 0.046) and absence of recurrent cephalosporins before diagnosis (*OR *= 0.05, 95% *CI*: 0.00–0.73, *P* = 0.028) were associated with remission.

**Conclusions:**

Infants are more prone to PBB, with increased wheezing. Gram-negative bacilli infections are common in infants in northeast China. Older children with PBB should be carefully assessed, treated and followed up, particularly those with long duration of cough and poor response to antibiotic treatments.

## 1. Introduction

Protracted bacterial bronchitis (PBB) was first proposed as a new disease entity in 2006 (1) and is increasingly being recognized as a clinically significant pediatric condition. In recent years, PBB has been incorporated into the guidelines for pediatric chronic (≥ 4 consecutive weeks without significative variations) cough (2–5). Although the global PBB prevalence within the community remains unknown, PBB has been identified as one of the most frequent causes of chronic cough in children in Australia (1, 6) and Turkey (7, 8), with an incidence ranging from 11% to 41% of all patients presenting for chronic cough. In addition, Chinese clinicians lack PBB diagnosis awareness (9). PBB is mostly diagnosed clinically according to chronic wet cough and response to treatment (3, 5). The definitive diagnosis can be made by positive bacterial sputum or bronchoalveolar lavage (BAL) fluid cultures, also called microbiological-based PBB (3, 5). However, bronchoscopy is an invasive and not risk-free procedure, and is not feasible in the routine clinical setting. Therefore, bronchoscopy should not be considered as a routine part of the PBB diagnostic workout but could be performed in selected cases.

It is important to diagnose and manage PBB appropriately. PBB has an intrinsic causal connection with bronchiectasis (10–12). PBB represents the early phase of chronic suppurative lung disease (CSLD). Irreversible bronchiectasis and subsequent lung function decline are bad ends of the event, which should alert pediatricians. The predominant clinical PBB features are chronic wet cough (≥4 weeks) due to endotracheal bacterial infections and clinical improvement after 2–4 weeks of appropriate antibiotic treatment without alternative causes for cough (3). However, the proposed criteria for PBB are non-specific and may lead to substantial misdiagnosis (13–15). Age-specific PBB incidence has been reported in a multi-center study on chronic cough in children, with a high incidence in younger children (6). Immune function and airway anatomy in younger and older children are different. However, few studies have evaluated age-specific clinical PBB features and outcomes in children.

*Haemophilus influenzae*, *Streptococcus pneumoniae*, and *Moraxella catarrhalis* are the top three reported causative PBB organisms (12, 16–23). There was a difference in PBB pathogen distribution in western countries and different areas of China (19–23), which may be due to different choices of antibiotics and different environments (22). In Chinese studies, more than 30% of PBB cases were diagnosed clinically without positive bacterial cultures, leading to misestimation of pathogen distributions (19, 21, 22). Local pathogen distribution and clinical features of PBB can help clinicians to diagnose and treat PBB (9). However, the PBB's pathogen distribution remains unclear in northeast China. Consequently, we aimed to investigate the clinical and etiological features and outcomes of children of different ages with PBB in northeast China.

## 2. Methods

### 2.1. Study subjects

A retrospective analysis was conducted using electronic medical records from the Shengjing Hospital of China Medical University's Pediatric Pulmonary Department between January 2017 and December 2021. Children aged <14 years with PBB diagnosis based on microbiologic assessment were enrolled in this study. The diagnostic criteria of microbiological-based PBB included (3, 24): (1) continuous wet or productive cough for more than 4 weeks (2); positive BAL fluid or sputum bacterial culture [>10^4^ colony-forming units (CFU)/mL] (3); resolution of cough following a 2–4-week course of appropriate antibiotic treatments; and (4) absence of alternative etiology for the wet or productive cough, like rhinitis, primary ciliary dyskinesia (PCD), cystic fibrosis (CF), etc. Premature infants and children with severe cardiac abnormalities, neuromuscular disease, gross neurodevelopmental delay, interstitial lung disease, confirmed severe lung disease, fever within 2 weeks, and incomplete original medical records were excluded. The children were divided into three age groups: <1 year (infants), 1–5 years (younger children) and ≥6 years (older children). This study was approved by the Ethics Committee of the Shengjing Hospital of China Medical University (No. 2022PS870K). The need for informed consent was waived off as data were anonymized.

### 2.2. Demographic and clinical data

Demographic data and clinical characteristics were collected retrospectively. Clinical manifestations included current cough (course, phase, and regularity), parent-reported wheezing and stridor and auscultation of the lung (moist rale, wheezing rale, or phlegm sound). Chest x-rays and chest computed tomography scans were also reviewed.

### 2.3. Microbiological and cellular analysis

BAL fluid was obtained for microbiological and cellular analysis. The flexible bronchoscopy (Olympus, Tokyo, Japan) was performed under moderate sedation using midazolam premedication with local anesthesia. Pediatric-type bronchoscopes were used according to the patient's age. Firstly, macroscopic evaluations were made by a trained pediatric pulmonologist, including tracheobronchial anatomy, kinesis, mucus secretion, and mucosal inflammation. After that, BAL fluid was sampled according to the American Thoracic Society guideline (25). Three equal sterile saline solutions (1 ml/kg, maximum ≤20 ml) were instilled into the most-affected bronchus indicated by radiology and/or bronchoscopy or into the right middle lobe in cases with diffuse abnormalities. The first BAL fluid aliquot was collected for microbiological analysis and processed as quickly as possible to avoid contamination. BAL fluid cultures for respiratory bacteria at densities ≥10^4^ CFU/mL were considered positive (3, 24, 25). BAL fluid polymerase chain reaction (PCR) assays were performed to detect respiratory syncytial virus, adenovirus, cytomegalovirus, influenza virus A and B, and *Mycoplasma pneumoniae*. The other two aliquots were sampled for differential cell counts.

In some infants and younger children, bronchoscopy was not performed; however, induced sputum samples were obtained for microbiological analysis. Before sputum suction, *β*_2_-agonist was administered using a nebulizer to prevent bronchospasm. After, 4.5% hypertonic saline was inhaled for 10 min using a facemask with mixed oxygen flow. Each quadrant of the chest's posterior portion was percussed gently 5–10 times during nebulization. A sterile mucus-extracting catheter attached to a suction device was inserted through the nose into the posterior nasopharynx. Suction was performed after the catheter was in place, and sputum was collected into a sterile trap. Suction was stopped during catheter withdrawal to prevent contamination. The catheter was flushed with 5 ml sterile saline at the end of the procedure, and the samples were immediately sent to the laboratory for analysis.

### 2.4. Treatment strategies and outcomes

Treatment strategies before hospitalization were recorded, including the use of cephalosporins, macrolides, inhaled corticosteroids, and systemic corticosteroids. During hospitalization, appropriate antibiotics were selected according to the bacterial culture and antibiotic sensitivity testing results, and administrated intravenously. Once the cough improved, they were discharged and placed on the corresponding oral antibiotics for 2–4 weeks. Outpatient clinic follow-up medical records were reviewed. If the records were missing, we followed up with caregivers *via* phone calls. The subject was excluded if follow-up information was unavailable. Remission was defined as the completely resolution of cough by 2-weeks of antibiotics treatments.

### 2.5. Statistical analysis

The data distributions were assessed using the Shapiro–Wilk test. Normally-distributed variables were expressed as the mean ± standard deviation and compared using one-way ANOVA. Non-normally distributed variables were expressed as the median [interquartile range (IQR)] and compared using the Kruskal–Wallis *H*-test. Categorical variables were expressed as percentages and compared using the *χ^2^* test (or Fisher's exact test in cases of expected frequencies <5). Post-hoc analysis was performed with the Bonferroni test. Factors associated with PBB remission during follow-up were examined using logistic regression. Two-tailed *P*-values <0.05 were considered statistically significant. Statistical analysis was performed using SPSS version 20.0 (IBM Corp., Armonk, NY).

## 3. Results

### 3.1. Clinical characteristics of PBB children by age

During the study period, 74 children were hospitalized for chronic wet cough and underwent bacterial cultures, whose cough improved after antibiotic treatments. Of these, 31 with positive BAL fluid bacterial cultures and 14 with positive sputum bacterial cultures were enrolled. The clinical characteristics are shown in Table 1. The median age was 0.9 years, ranging from 0.16 to 13.11 years, with 24 (53.3%) infants (<1 year) and 37 (82.2%) children (≤5 years). Infants with PBB were more often boys (66.7%), whereas older children (≥6 years) were more often girls (87.5%). The cough duration increased with age, with the longest occurring in the older children (*P* = 0.038). No differences in cough regularities were found among the age groups. Paroxysmal cough was most common (62.2%) type of cough, which disappeared after sputum expectoration. In addition, rattling sounds in the chest, pertussis-like cough, and stridor were only observed in infants. Over half (51.1%) of children with PBB had parent-reported wheezing, particularly children ≤5 years. During the physical examination on admission, moist rale, wheezing rale, and phlegm sounds were recorded in 17 (37.8%), 19 (42.2%), and 14 (30.4%) patients, respectively. A higher rate of wheezing rales was found in infants than that in the older children (*P* = 0.016).

**Table 1:** Clinical characteristics of PBB children stratified by ages.

Characteristics	Total	<1 year	1–5 years	≥6 years	*P-*value
	(*n* = 45)	(*n* = 24)	(*n* = 13)	(*n* = 8)	
Gender (male %)	23 (51.1%)	16 (66.7%)	6 (46.2%)	1 (12.5%)^a^	0.024
Symptoms					
Cough					
Duration (months)	1.5 (1.0–4.0)	1.0 (1.0–2.8)	2.0 (1.3–6.0)	3.8 (1.1–19.5)	0.038
Regularity					
Morning	3 (6.7%)	1 (4.2%)	1 (7.7%)	1 (12.5%)	0.747
Night	12 (26.7%)	6 (25.0%)	2 (15.4%)	4 (50.0%)	0.254
Post-activity	8 (17.8%)	3 (12.5%)	4 (30.8%)	1 (12.5%)	0.388
Paroxysmal	28 (62.2%)	16 (66.7%)	8 (61.5%)	4 (50.0%)	0.715
Irregular	16 (35.6%)	9 (37.5%)	5 (38.5%)	2 (25.0%)	0.837
Pertussis-like	4 (8.9%)	4 (16.7%)	0 (0.0%)	0 (0.0%)	0.277
Rattling in the chest	5 (11.1%)	5 (20.8%)	0 (0.0%)	0 (0.0%)	0.094
Stridor	4 (8.9%)	4 (16.7%)	0 (0.0%)	0 (0.0%)	0.277
Parent-reported wheezing	23 (51.1%)	15 (62.5%)	8 (61.5%)	1 (12.5%)^a^	0.046
Nasal symptoms	12 (26.7%)	2 (8.3%)	7 (53.8%)^a^	3 (37.5%)	0.007
Auscultation of lung					
Moist rales	17 (37.8%)	10 (41.7%)	4 (30.8%)	3 (37.5%)	0.915
Wheezing rales	19 (42.2%)	15 (62.5%)	3 (23.1%)	1 (12.5%)^a^	0.016
Phlegm sounds	14 (30.4%)	10 (41.7%)	3 (23.1%)	1 (12.5%)	0.289

Data are expressed as median (IQR) or n (%). *P*-values comparison among the age groups using the Kruskal–Wallis test or *χ^2^* test (Fisher's exact test, followed by post-hoc analysis with Bonferroni).

^a^ *P *< 0.05 as compared to the <1 year age group.

### 3.2. Radiography and bronchoscopy findings of PBB children by age

All children included underwent chest x-ray and-or high-resolution computed tomography (HRCT)-scan. Thirty-six children received HRCT-scan and the findings are summarized in Table 2. The chest x-ray lacked specific findings, showing only increased lung markings and patchy consolidations. 22.2% of HRCT-scans were reported as normal. The most common abnormality reported was bronchial wall thickening (55.6%), followed by patchy consolidation (36.1%), and peribronchial inflammation (22.2%). Older children had the highest proportion of bronchial wall thickening (*P* = 0.043), and infants had the highest proportion of patchy consolidation of the right upper and lower lobe (*P* = 0.041).

**Table 2:** Radiography and bronchoscopy findings of PBB children stratified by ages.

Findings	Total	<1 year	1–5 years	≥6 years	*P-*value
	(*n* = 45)	(*n* = 24)	(*n* = 13)	(*n* = 8)	
HRCT findings	*n* = 36	*n* = 15	*n* = 13	*n* = 8	* *
Normal	8 (22.2%)	3 (20.0%)	4 (30.8%)	1 (12.5%)	0.678
Bronchial wall thickening	20 (55.6%)	5 (33.3%)	8 (61.5%)	7 (87.5%)^a^	0.043
Segmental hyperinflation	5 (13.9%)	1 (6.7%)	3 (23.1%)	1 (12.5%)	0.505
Peribronchial inflammation	8 (22.2%)	3 (20.0%)	3 (23.1%)	2 (25.0%)	1.000
Patchy consolidation	13 (36.1%)	7 (46.7%)	3 (46.7%)	3 (37.5%)	0.448
Right middle lobe and lingula	4 (11.1%)	0 (0.0%)	2 (15.4%)	2 (25.0%)	0.100
Right upper and lower lobe	9 (25.0%)	7 (46.7%)	1 (7.7%)	1 (12.5%)	0.041
Bronchoscopy findings	*n* = 31	*n* = 11	*n* = 12	*n* = 8	
Macroscopic findings					
Thick mucus secretion	8 (25.8%)	1 (9.1%)	5 (41.7%)	2 (25.0%)	0.263
Mucosal edema and hyperemia	17 (54.8%)	7 (63.6%)	6 (50.0%)	4 (50.0%)	0.813
Mucosal folds	5 (16.1%)	0 (0.0%)	3 (25.0%)	2 (25.0%)	0.215
Bronchial stenosis	7 (22.6%)	2 (18.2%)	3 (25.0%)	2 (25.0%)	1.000
Tracheobronchial malacia	4 (12.9%)	4 (36.4%)	0 (0.0%)	0 (0.0%)	0.013
BAL fluid differential cell counts					
Neutrophil (%)	31.0 (11.8–70.0)	17.0 (10.0–54.0)	31.5 (14.5–75.8)	70.0 (29.0–89.0)^a^	0.015
Macrophage (%)	47.5 (7.5–63.0)	52.0 (26.0–63.0)	54.0 (10.0–72.8)	5.0 (3.0–45.0)^a^	0.020
Lymphocyte (%)	8.5 (4.0–11.0)	10.0 (5.0–19.0)	7.0 (3.5–9.8)	10.0 (2.0–25.0)	0.098
Eosinophil (%)	0.0 (0.0–0.0)	0.0 (0.0–0.0)	0.0 (0.0–0.0)	0.0 (0.0–0.0)	0.193
Epithelial (%)	2.5 (0.0–10.8)	9.0 (2.0–16.0)	0.5 (0.0–7.3)	0.0 (0.0–10.0)	0.087
Positive bacterial cultures (top three)	*n* = 45	*n* = 24	*n* = 13	*n* = 8	
* Haemophilus influenzae*	19 (42.2%)	7 (29.2%)	6 (46.2%)	6 (75.0%)	0.088
* Streptococcus pneumoniae*	10 (22.2%)	3 (12.5%)	6 (46.2%)	1 (12.5%)	0.066
* Klebsiella pneumoniae*	9 (20.0%)	9 (37.5%)	0 (0.0%)^a^	0 (0.0%)	0.008

Data are expressed as median (IQR) or *n* (%). *P*-values comparison among the age groups using the Kruskal–Wallis test or *χ^2^* test (Fisher's exact test followed by post-hoc analysis with Bonferroni). BAL, bronchoalveolar lavage; HRCT, high resolution computed tomography.

^a^ *P *< 0.05 as compared to the <1 year age group.

Bronchoscopy was performed in 31 cases; the findings are summarized in Table 2. The most common macroscopic finding was mucosal edema and hyperemia (54.8%), followed by thick mucus secretion (25.8%), bronchial stenosis (22.6%), mucosal folds (16.1%), and tracheobronchial malacia (12.9%). There were no significant differences among the age groups regarding macroscopic findings, except for a higher proportion of tracheobronchial malacia in infants (*P* = 0.013). Older had a higher BAL fluid neutrophil percentage (*P* = 0.015) and lower BAL fluid macrophage percentage (*P* = 0.020) than infants. There were no significant differences in lymphocyte and eosinophil percentages among the age groups.

### 3.3. Microbiological findings of PBB children by age

**Figure 1:**
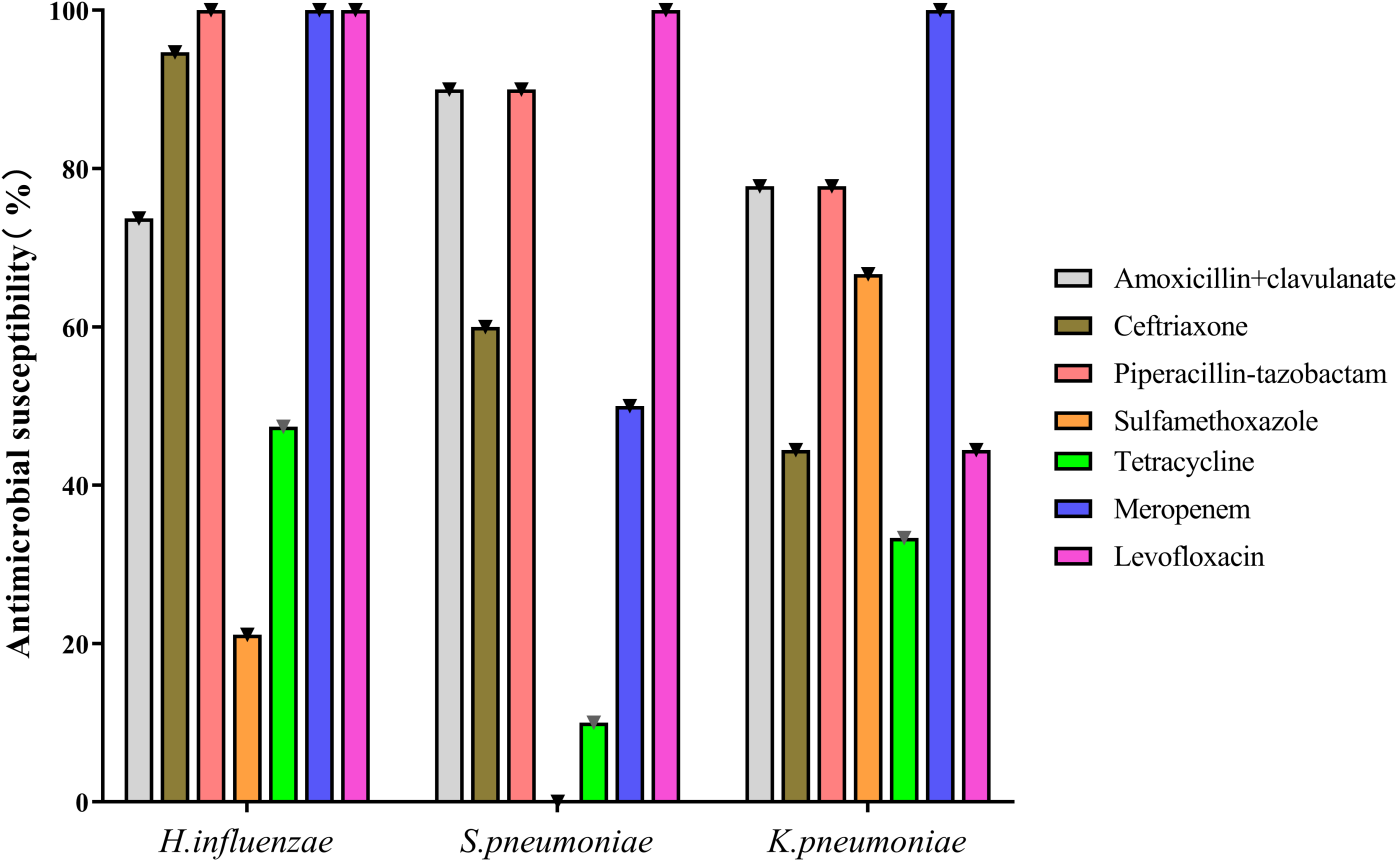
In *vitro* antibiotic sensitivity of *H. influenzae*, *S. pneumoniae*, *and K. pneumoniae*.

Bacteria were cultured from BAL fluid in 31 cases, with sputum induction in only 14 (Table 2 and Supplementary Table S1). The most commonly cultured bacteria were *H. influenzae* (42.2%), followed by *S. pneumoniae* (22.2%) and *Klebsiella pneumoniae* (20.0%). *H. influenza* was the most common microorganism in younger (46.2%) and older (75.0%) children, and *K. pneumoniae* was the most common in infants (42.2%). *K. pneumoniae*, *Escherichia coli*, *Enterobacter aerogenes*, *Enterobacter cloacae*, *Serratia marcescens*, and *Pseudomonas maltophilia* were only detected in infants; no differences were found among the age groups except for *K. pneumoniae*. More than one bacterium was cultured in four children. The *in vitro* antibiotic sensitivity results of the most common three bacteria are shown in Figure 1. *Mycoplasma pneumoniae*, respiratory syncytial virus, and cytomegalovirus were also detected by PCR assays in 20.0%, 6.7%, and 4.4% of patients, respectively.

### 3.4. Treatments and follow-up

Before hospitalization, 31 (68.9%) children received macrolides, and only two (4.4%) had a 2-week cephalosporin course (Supplementary Table S2). About half (53.3%) of the children received inhaled or systemic corticosteroids, with a higher rate in infants (*P* = 0.039). After PBB diagnosis, the children were treated with amoxicillin/clavulanate, cephalosporins, and carbapenems according to antibiotic sensitivity. Over half (66.7%) of the patients were symptom-free after 2 weeks of antibiotic use. Infants had a higher remission rate than older children (*P* = 0.009). Four cases with right middle lobe and lingula infiltrate required 4–5 weeks of antibiotic use to improve. Logistic regression analysis showed that shorter cough duration (*OR *= 0.58, 95% *CI*: 0.34–0.99, *P* = 0.046) and absence of recurrent cephalosporins before diagnosis (*OR *= 0.05, 95% *CI*: 0.00–0.73, *P* = 0.028) were associated with remission (Table 3). Age at diagnosis did not contribute significantly to the model.

**Table 3:** Logistic regression analysis of predictors of “remission” of PBB children.

Variables	*OR*	95% *CI* (lower–upper)	*P*-value
Age (year)	0.99	0.97–1.01	0.186
Duration of cough (month)	0.58	0.34–0.99	0.046
Recurrent cephalosporins before hospitalization	0.05	0.00–0.73	0.028

CI, confidence interval; OR, odds ratio.

At 1-year follow-up, only five (11.1%) children relapsed and required intermittent antibiotics, most common in older children. Among them, three had previous severe *Mycoplasma pneumoniae* or adenovirus pneumonia, and relapsed three or more times per year. Four children were diagnosed with bronchiectasis through HRCT examinations during follow-up, and PCD and CF were excluded by genetic testing. During the 1-year follow-up period, two infants and five younger children had recurrent wheezing without chronic wet cough, three of whom were diagnosed with asthma according to a positive bronchodilation test with excellent responses to inhaled corticosteroids.

## 4. Discussion

This retrospective analysis describes the clinical features and outcomes of PBB confirmed microbiologically in children stratified by age. Consistent with previous studies (6, 8, 12, 15, 17), we found that the incidence of PBB vary with age, with that being highest in infants. Wheezing was a common symptom in infants with PBB, often misdiagnosed as asthma and treated with corticosteroids. PBB was frequently overlooked in the differential chronic wet cough diagnosis, leading to inappropriate choices and inappropriately short antibiotic courses. Gram-negative bacilli were common PBB pathogens in infants, particularly *K. pneumoniae* and *H. influenzae*. There are several appropriate antibiotics to eradicate these bacteria but amoxicillin/clavulanate should be the first choice. In addition, although older children made up a modest portion of PBB, they should be carefully assessed, treated, and followed up because they are at a high risk of longer cough before treatment and it is harder to gain eradication in these patients.

In the present study, the median age of PBB patients was 0.9 years, ranging from 0.16 to 13.11 years, with 24 (53.3%) infants. In consistent with this, Wang et al. reported 50 PBB patients with median age of 10 months (14). Donnelly et al. found that 59% of children had symptoms of PBB before the age of 2 years (26). In a cohort of chronic wet cough, over half of children aged less than 2 years were diagnosed as PBB and the authors suggested that the younger the age group, the more likely PBB was found (6). However, the median age of PBB patients was 2.3 (1.2–2.8) and 1.7 (0.6–7.4) years, in the studies by Baines et al. (27) and Marsh et al. (28), respectively, which was slightly higher than that in our study. The congenital and genetic abnormalities, like PCD, CF, immunodeficiencies, anatomical anomalies, etc. are more common seen in infants and can also cause recurrent infections of lower airway. Before the diagnosis of PBB in infants, careful evaluation should be performed.

In the present study, 15 (62.5%) infants had parent-reported wheezing and wheezing rales on auscultation, which was significantly higher than that of older children. Only two infants had wheezing episodes during the 1-year follow-up. Consistent with the present study, Wurzel et al. reported that 81% of children with PBB had parent-reported wheezing, which resolved after two weeks of antibiotic therapy and could not be explained by coexistent asthma (17). Donnelly et al. highlighted that children with PBB did not have a “wheeze” but a rattling sound reflective of airway secretions (26). However, in the present study, parent-reported wheezing and wheezing rales on auscultation were consistent in infants, and rattling sounds were only present in five infants. The high prevalence of auscultatory wheezing in our study may be related to the relatively high proportion of infants. Wang et al. reported 82% of PBB patients younger than 3-year-old had auscultatory wheezing (14). The high frequency of wheezing in infants may be due to relatively narrow airway lumen, which is susceptible to obstruction by mucus, mucosal edema, mucosal folds, airway malacia, etc. PBB is an overlooked disease and the parent-reported wheezing led to corticosteroids prescription (14, 17, 26). In our study, 42.2% of children received inhaled corticosteroids, and 20.0% received systemic corticosteroids because of a suspected diagnosis of asthma in primary settings. The high proportion of infants receiving ICS can figure malpractice according to GINA indications about ICS (29). A diagnosis of asthma should be nearly never performed in an infant and performed with caution in a toddler because they may present recurrent episodes of viral wheezing, which is a completely different condition.

PBB is frequently misdiagnosed (13–15). PBB can be diagnosed clinically or microbiologically (3). Positive BAL fluid culture is high-quality evidence of PBB; however, it is impractical for all children with a chronic wet cough to undergo bronchoscopy (3). In the present study, 13 infants were diagnosed using positive induced sputum cultures, which may be more feasible for infants, reducing adverse events and bronchoscopy costs (30). Consistent with previous studies (14, 23, 26), the main findings of chest radiograph were bronchial wall thickening, patchy consolidation, and peribronchial inflammation. In our study, segmental hyperinflation was detected in 5 (13.9%) PBB patients, which was only reported by Guan et al. (23) in 5 (4.9%) PBB patients. This uncommon finding might be caused by partial obstruction of airway caused by mucus, mucosal edema, mucosal folds, etc. The relatively high prevalence of segmental hyperinflation in our study may be due to that the chest CT were performed during infective processes. Chest radiograph did not contribute to PBB diagnosis and management, which should be performed only in children at risk of bronchiectasis by low dose CT instead of the HRCT as recommended by the guideline (3). If performed, chest CT should be three or four months after the symptom's resolution, but not during infective processes. Because inflammation related bronchial ectasis are frequently reversable, while bronchiectasis are not reversable by definition (31).

PBB is always diagnosed clinically according to the typical chronic wet cough and response to appropriate antibiotic treatment (3). In the present study, the failure of diagnosis before hospitalization might be caused by the inappropriate choices and short courses of antibiotics. Macrolides were used in 68.9% of children, and *in vitro* antibiotic sensitivity testing showed that all bacteria were resistant to macrolides. A survey of 1,022 Chinese pediatricians found that 23.5% chose macrolides as the first-line antibiotic for PBB (9). Although 26 (57.8%) children received cephalosporins before hospitalization, 24 had a course of 5–10 days. Clinicians frequently overlook PBB in the differential diagnosis, particularly general pediatricians (9); as a result, they are more likely to modify the antibiotic therapy than prolong it. The high incidence of wheezing also confounded the diagnosis, particularly in infants. Most guidelines recommend two weeks of antibiotics, and a longer antibiotic course of four weeks was also suggested for patients with poor response and without any other specific cough pointers (3, 24). A recent randomized controlled trial demonstrated that compared to a 2-week amoxicillin/clavulanate course, a 4-week course had little advantage in achieving clinical cure but led to a longer cough-free period (32). Results of present study demonstrated that longer duration of cough and recurrent cephalosporins were risk factors for poor improvement. Therefore, appropriate antibiotics and enough courses are important for PBB diagnosis, and a longer course and careful assessment are warranted for children with longer cough duration.

In the present study, *H. influenzae, S. pneumoniae,* and *K. pneumoniae* were the most common bacteria implicated in PBB. In contrast to studies in western countries (12, 16–18, 22), *M. catarrhalis* was isolated only in 6.7% of children, consistent with studies in China (19, 21, 22). Another interesting finding was that infants with PBB had various microorganisms dominated by gram-negative bacilli, particularly *K. pneumoniae* (37.5%). *K. pneumoniae* has been reported as a PBB pathogen in several Chinese researches (20, 23). The difference in the bacterial spectrum may be due to the region-specific bacteria distribution and antibiotic use before bacterial cultures (22). Wurzel et al. showed that only 10% of children received antibiotics before bronchoscopy; in contrast, almost all children received antibiotics in our study (11, 17). In addition, a recent study found that *K. pneumoniae* (35.3%) was the most frequent isolate from home nebulization sets, resulting in its dominance in infants (33). Other gram-negative bacilli, like *E. coli*, *E. aerogenes*, *E. cloacae*, *S. marcescens*, and *P. maltophilia*, were only present in infants. In *vitro* antibiotic sensitivity analysis showed that more than 70% of the three common isolated bacteria were sensitive to amoxicillin/clavulanate. Amoxicillin/clavulanate is still the first choice antibiotic for PBB in children, although there are several appropriate antibiotics to eradicate these bacteria (3, 10, 34). Ceftazidime or carbapenems can be tested in infants with *Enterobacteriaceae* infections and poor response to amoxicillin/clavulanate. In the present study, the antibiotics were administrated intravenously during hospitalization and orally after discharge. Usually, PBB treatment can be started and carry on orally.

Consistent with previous studies (6, 8, 11, 12, 17), we found that older children made up a modest portion of PBB and should be carefully assessed, treated, and followed up because they are at high risk of developing bronchiectasis. In the present study, older children with PBB were more often girls, which is inconsistent with the observed gender preference in PBB (6, 11, 12, 17) but consistent with that of bronchiectasis (35, 36). King et al. reported 182 adult patients with bronchiectasis (35); 107 (59%) had developed a chronic productive cough in childhood, 80 (75%) of whom were female. Aksamit et al. reported 1,826 adult patients with bronchiectasis, 79% of whom were female (36). In addition, we found that older children had relatively longer cough duration, higher rate of *H. influenzae* infections, more bronchial wall thickening on HRCT, lower remission rate, and high relapse rate. *H. influenzae* in the BAL fluid correlate with the risk of developing bronchiectasis in children with PBB (11, 12). Moreover, four older children were diagnosed with bronchiectasis during follow-up. PBB, CSLD, and bronchiectasis were proposed to increase severity (10). Recent studies also demonstrated that PBB was associated with a future diagnosis of bronchiectasis (11, 12). Therefore, a detailed review should be carried out on children with long cough duration and poor response to adequate antibiotic treatment, including, if necessary, CT-scan, bronchoscopy, immune function, or genetic testing.

This study had several limitations due to its descriptive nature and retrospective design. Although the data were meticulously recorded, they were collected from electronic medical records, some of which were incomplete. A strong selection bias should be taken into the account, because in PBB most patients undergo clinical diagnosis and only very severe patients or patients with unusual clinical pictures undergo bronchoscopy. Our findings only covered the microbiological diagnostic PBB, which may be different from the clinical one. In younger children and infants, induced sputum is easy to collect and well tolerated, but is easily contaminated by upper airway secretions. While, induced sputum has been shown to be a useful alternative to BAL for the detection of lower airway pathogens in children with CF (37, 38). In addition, the follow-up periods were relatively short. It could not be evaluated whether infants with PBB could develop asthma during preschool or school age. Moreover, this single-center study could only reflect the local distribution of bacteria and antibiotic sensitivities. Our hospital is the National Regional Children's Medical Center Northeast, and the patients in our department cover most areas in the northeast of China; therefore, the present study's results could help diagnose and treat PBB in northeast China. Further prospective multi-center studies on both clinical and microbiological diagnostic PBB with longer follow-up periods are warranted.

In summary, infants are more prone to PBB and wheezing, which is often misdiagnosed as asthma. Gram-negative bacilli infections are common in infants, particularly *K. pneumonia*. Older children with PBB should be carefully assessed, treated and followed up, particularly those with long cough duration and poor response to adequate antibiotic treatments.

## Data Availability

The raw data supporting the conclusions of this article will be made available by the authors, without undue reservation.
