# Supplementary material for: Clinical features and pathogen distributions of microbiological-based protracted bacterial bronchitis in children of different ages in Northeast China
Source: Front Pediatr. 2023 Apr 21;11:1163014. doi: 10.3389/fped.2023.1163014 (PMC10162439; doi:10.3389/fped.2023.1163014)
Supplement: Supplementary file 1 [file Table1.docx]

Supplementary Material

# Supplementary Tables

## Supplementary Table 1. Microbiological findings of PBB children stratified by ages.

| **Microorganisms** | **Total**  **(n=45)** | **<1 year**  **(n=24)** | **1–5 years**  **(n=13)** | **≥6 years**  **(n=8)** | ***P-*value** |
| --- | --- | --- | --- | --- | --- |
| **Positive bacterial cultures** | | | | | |
| *Haemophilus influenzae* | 19 (42.2%) | 7 (29.2%) | 6 (46.2%) | 6 (75.0%) | 0.088 |
| *Streptococcus pneumoniae* | 10 (22.2%) | 3 (12.5%) | 6 (46.2%) | 1 (12.5%) | 0.066 |
| *Klebsiella pneumoniae* | 9 (20.0%) | 9 (37.5%) | 0 (0.0%)^a^ | 0 (0.0%) | 0.008 |
| *Moraxella catarrhalis* | 3 (6.7%) | 2 (8.3%) | 1 (7.7%) | 0 (0.0%) | 1.000 |
| *Enterobacter aerogenes* | 2 (4.4%) | 2 (8.3%) | 0 (0.0%) | 0 (0.0%) | 0.685 |
| *Enterobacter cloacae* | 2 (4.4%) | 2 (8.3%) | 0 (0.0%) | 0 (0.0%) | 0.685 |
| *Staphylococcus aureus* | 1 (2.2%) | 0 (0.0%) | 0 (0.0%) | 1 (12.5%) | 0.178 |
| *Escherichia coli* | 1 (2.2%) | 1 (4.2%) | 0 (0.0%) | 0 (0.0%) | 1.000 |
| *Serratia marcescens* | 1 (2.2%) | 1 (4.2%) | 0 (0.0%) | 0 (0.0%) | 1.000 |
| *Pseudomonas maltophilia* | 1 (2.2%) | 1 (4.2%) | 0 (0.0%) | 0 (0.0%) | 1.000 |
| ≥2 types of bacteria | 4 (8.9%) | 4 (16.7%) | 0 (0.0%) | 0 (0.0%) | 0.277 |
| ***Mycoplasma pneumoniae*** | 9 (20.0%) | 5 (20.8%) | 2 (15.4%) | 2 (25.0%) | 0.890 |
| **Virus** |  |  |  |  |  |
| Respiratory syncytial virus | 3 (6.7%) | 3 (12.5%) | 0 (0.0%) | 0 (0.0%) | 0.416 |
| Cytomegalovirus | 2 (4.4%) | 2 (8.3%) | 0 (0.0%) | 0 (0.0%) | 0.685 |
| **Coinfection** | 16 (35.6%) | 12 (50.0%) | 2 (15.4%) | 2 (25.0%) | 0.107 |

Data are expressed as n (%). *P*-values comparison among the age groups using the *χ^2^* test (Fisher’s exact test followed by post-hoc analysis with Bonferroni). ^a^ *P*<0.05 as compared to the <1 year age group.

## Supplementary Table 2. Treatments and outcomes of PBB children stratified by ages.

| **Items** | **Total**  **(n=45)** | **<1 year**  **(n=24)** | **1–5 years**  **(n=13)** | **≥6 years**  **(n=8)** | ***P-*value** |
| --- | --- | --- | --- | --- | --- |
| **Treatments before hospitalization** | | | | | |
| Cephalosporins | 26 (57.8%) | 12 (50.0%) | 8 (61.5%) | 6 (75.0%) | 0.516 |
| Cephalosporins ≥2 weeks | 2 (4.4%) | 1 (4.2%) | 0 (0.0%) | 1 (12.5%) | 0.406 |
| Macrolides | 31 (68.9%) | 17 (70.8%) | 9 (69.2%) | 5 (62.5%) | 0.912 |
| Corticosteroids | 24 (53.3%) | 17 (70.8%) | 5 (38.5%) | 2 (25.0%) | 0.039 |
| Inhaled corticosteroids | 19 (42.2%) | 13 (54.2%) | 4 (30.8%) | 2 (25.0%) | 0.257 |
| Systemic corticosteroids | 9 (20.0%) | 7 (29.2%) | 2 (15.4%) | 0 (0.0%) | 0.183 |
| **Treatment after diagnosis** | | | | | |
| Amoxicillin/clavulanate | 24 (53.3%) | 12 (50.0%) | 7 (53.8%) | 5 (62.5%) | 0.921 |
| Cephalosporin | 11 (24.4%) | 6 (25.0%) | 4 (30.8%) | 1 (12.5%) | 0.732 |
| Carbapenem | 7 (15.6%) | 4 (16.7%) | 1 (7.7%) | 2 (25.0%) | 0.557 |
| Linezolid | 4 (9.1%) | 2 (8.7%) | 1 (7.7%) | 1 (12.5%) | 1.000 |
| **Length of hospital stay** | 10.4±3.9 | 11.0±3.8 | 9.6±2.6 | 10.3±2.6 | 0.609 |
| **Outcomes** |  |  |  |  |  |
| Follow-up after 1 month |  |  |  |  |  |
| Remission | 30 (66.7%) | 20 (83.3%) | 8 (61.5%) | 2 (25.0%)^a^ | 0.009 |
| Improve | 15 (33.3%) | 4 (16.7%) | 5 (38.5%) | 6 (75.0%)^a^ | 0.009 |
| Follow-up after 1 year |  |  |  |  |  |
| Relapse | 5 (11.1%) | 1 (4.2%) | 1 (7.7%) | 3 (37.5%) | 0.059 |
| Relapse ≥ 3 times | 3 (6.7%) | 0 (0.0%) | 1 (7.7%) | 2 (37.5%) | 0.050 |
| Recurrent wheezing | 7 (14.3%) | 2 (8.3%) | 5 (33.3%) | 0 (0.0%) | 0.042^*^ |

Data are expressed as mean ± SD or n (%). *P*-values comparison among the age groups using the one-way ANOVA or *χ^2^* test (Fisher’s exact test followed by post-hoc analysis with Bonferroni). ^a^ *P*<0.05 as compared to the <1 year age group.
